# *Haemophilus parainfluenzae* as a marker of the upper respiratory tract microbiota changes under the influence of preoperative prophylaxis with or without postoperative treatment in patients with lung cancer

**DOI:** 10.1186/s12866-016-0679-6

**Published:** 2016-04-06

**Authors:** Urszula Kosikowska, Anna Biernasiuk, Paweł Rybojad, Renata Łoś, Anna Malm

**Affiliations:** Department of Pharmaceutical Microbiology with Laboratory for Microbiological Diagnostics, Medical University of Lublin, Lublin, Poland; Department of Thoracic Surgery, Medical University of Lublin, Lublin, Poland; Department of Pharmaceutical Microbiology with Laboratory for Microbiological Diagnostics, Medical University of Lublin, Chodzki Str. 1, Lublin, 20-093 Poland

**Keywords:** *Haemophilus parainfluenzae*, *Candida* spp., Respiratory microbiota, Lung cancer, Perioperative prophylaxis, Postoperative treatment

## Abstract

**Background:**

Haemophili are representative microbiota of the upper respiratory tract. The aim of this study was to assess the effects of perioperative antimicrobial prophylaxis and/or postoperative treatment on *Haemophilus parainfluenzae* prevalence, and antimicrobial sensitivity in short-term hospitalized patients with lung cancer who underwent surgery.

**Results:**

Samples were collected from 30 short-term hospitalized patients with lung cancer and from 65 healthy people. The nasal and throat specimens were taken twice from each patient: before (EI, Examination I), on the fourth/fifth day (EII, Examination II) after surgery, and once from healthy people. The isolates identification and antimicrobial susceptibility were detected by routine diagnostic methods. *H. parainfluenzae* was found in throat specimens of 42/65 (64.6 %) healthy people, while in 19/30 (63.3 %) lung cancer patients in EI (*p* = 0.6203) and in 13/30 (43.3 %) ones in EII (*p* = 0.0106). Neither the disease itself nor short-term hospitalization with perioperative prophylaxis alone affected *H. parainfluenzae* prevalence in EII, while perioperative prophylaxis with postoperative treatment significantly decreased its colonization in EII. The differences in the number of patients colonized by *Candida* spp. in EI and in EII were observed (*p* = 0.0082).Totally, 23/58 (39.7 %) of *H. parainfluenzae* isolates were resistant mainly to beta-lactams; among 11 ampicillin-resistant isolates only 3 were beta-lactamase positive.

**Conclusions:**

The antimicrobial perioperative prophylaxis together with postoperative treatment may disturb the composition of the airways microbiota represented by *H. parainfluenzae*, in addition to selecting the resistant strains of bacteria and promoting yeasts colonization in lung cancer patients undergoing surgery.

## Background

The microbiota is very important in the life style conditions and health safety [1–3]. Among numerous microorganisms of the upper respiratory tract microbiota, *Haemophilus* spp. may be common and representative. Haemophili play a role in preventing the establishment of potential pathogens and are very important for the proper functioning of the human body, including defense mechanisms [4, 5]. The changes in the composition of microbiota can cause various dysfunctions of the protective barrier of the airways and may contribute to an increase in mucosal colonization by pathogenic microorganisms including the environmental ones, eg hospital microflora [6–8]. Additionally, it can be regarded as a reservoir of opportunistic pathogens. The microbiota disturbance may predispose to other bacteria or fungi colonization and respiratory infections [6, 9]. According to some studies, fungi like *Candida* spp. or *Aspergillus* spp., Gram-negative rods (mainly *Enterobacteriaceae* and *Pseudomonadaceae*) and Gram-positive bacteria (eg *Staphylococcus* spp. or *Streptococcus* spp.) are the important etiological factors of diseases in patients with lung cancer [9, 10].

Haemophili, mainly *Haemophilus influenzae* and occasionally *Haemophilus parainfluenzae*, can cause a variety of invasive, chronic or recurrent diseases [2, 11, 12]. *H. parainfluenzae* is increasingly recognized as an opportunistic pathogen responsible for various infections [13, 14], including the respiratory tract infections [15–17], endocarditis [18–20], bacteraemia or even sepsis [21, 22]. Qualitative and quantitative changes in resident or transient members of the respiratory tract microbiota, eg *Haemophilus* spp., may be a risk factor of endogenous infections, which are an important medical problem among several groups of patients, eg immunocompromised patients.

Surgery remains the most effective treatment in lung cancer [23]. As patients with neoplastic changes already have the weakened defense mechanisms, prophylactic antibiotic treatment is usually administrated before, during, or after diagnostic and therapeutic procedures [24–27]. During hospitalization, both the patient’s microbiota and its susceptibility to antimicrobials may undergo qualitative and quantitative changes. It depends on the environmental conditions and the type (prophylaxis and/or antimicrobial treatment) as well as the length of an antibiotic application.

The present study seeks to determine the prevalence and antimicrobial sensitivity of the haemophili species (mainly *H. parainfluenzae*) as the upper respiratory tract microbiota, depending on the perioperative prophylaxis and/or postoperative treatment with commonly used antibiotics in short-term hospitalized lung cancer patients who underwent surgery.

## Methods

### Patients

Thirty patients aged 43-75 years old (average 62.1) with lung cancer who were admitted to the Department of Thoracic Surgery of Medical University of Lublin (between February 2011 and March 2012) were included in the study. Lung cancer was determined before surgery on the basis of an examination of specimens obtained during bronchoscopy, thin needle biopsy or sputum cytology. At the beginning of the sampling, none of the patients qualified for the study had any clinical evidence of viral or bacterial airways infections (normal body temperature and leukocyte count). Within 30 days before being admitted to hospital, no patients had taken any antimicrobial agents or drugs influencing the immunological system. Moreover, they neither had any blood transfusions nor suffered from an allergic disease. All patients with resectable lung cancer were subjected to microbiological studies in a comparable time both before surgery, which was performed on the day of hospital admission and before antibiotic treatment (EI, Examination I), and four or five days after the surgery accompanied by preoperative antimicrobial prophylaxis with or without postoperative antibiotic treatment (EII, Examination II). All patients were operated on one or two days after hospital admission. Each patient received preoperative antimicrobial prophylaxis with beta-lactams (cefuroxime or cefazolin) with or without amikacin. For prophylactic purposes the commonest route of administration was one single dose of antibiotics; the drug should be given not later than about 0.5 hour before the commencement of surgery; in prolonged surgery re-dosing at 4 hour’s intervals is indicated. Moreover, 15 patients had beta-lactams treatment and 1 patient had beta-lactams with amikacin treatment extended onto the postoperative period for the next four or five days.

The control (reference) group was established out of 65 healthy volunteers aged 19-75 years old (average 45.3 years). All patients agreed to participate in the research. The study was approved by the Ethical Committee of Medical University of Lublin (No. KE-0254/75/2011).

### Microbiological assay

The specimens – one swab from throat and two swabs from nose (from the left and right nostrils independently) – were taken from each lung cancer patient twice: first on the day of hospital admission and before antibiotic treatment (EI) and then on the fourth/fifth day after thoracic surgery (EII). A total of 180 specimens were taken from 30 patients with lung cancer including 60 swabs from the throat (in EI – 30, and in EII – 30) and 120 nasal swabs (in EI – 60, and in EII – 60). Additionally, 65 swabs were taken from healthy people’s throat once.

The specimens were taken by means of sterile cotton swabs and they were immediately placed onto the appropriate nonselective medium (5 % sheep blood agar) and the selective medium for haemophili (*Haemophilus* chocolate agar, HAEM, bioMérieux, France), Gram-negative rods (McConkey agar, Oxoid, England), and for fungi (Sabouraud dextrose agar, bioMérieux, France; BBL Chromagar *Candida*, Becton Dickinson and Company, USA).

The blood agar, McConkey and Sabouraud medium were incubated under aerobic conditions for 18-48 hours at 35 °C. After incubation, colonies from both McConkey agar and Sabouraud dextrose agar were selected and cultured on the agar media for bacteria and for fungi for identification of the isolates. The presence of bacteria and yeasts in the upper airways in at least one sample was considered as colonization. The strains of Gram-negative bacteria and fungi were identified using biochemical microtests (bioMérieux, France) - API 20E (for *Enterobacteriaceae* family) and API 20NE (for *Pseudomonadaceae* family) or API ID 32C (for *Candida* spp.). The ability of *Candida* strains to produce hyphae, pseudohyphae or chlamydospores was also evaluated.

The HAEM medium for haemophili was incubated in the atmosphere with an increased 5 % CO_2_ concentration (appropriate for microaerophilic bacteria) for 18-48 hours at 35 °C. After incubation, the growth of bacteria in the form of individual colonies or from abundant to a very abundant number of morphologically different colonies on Chocolate agar was observed. For the initial identification of isolated haemophili, morphological characteristics of the colonies growing on HAEM agar and the requirements for hemin (X factor) and nicotinamide adenine dinucleotide (V factor) on TSA (Tripticasein Soy Lab-Agar, Biocorp, Poland) medium with diagnostic discs DD3 (X factor), DD4 (V factor), DD5 (both X and V factors) obtained from Oxoid (England) were determined. Biochemical identification of isolates was carried out using the API NH microtest (bioMérieux, France). The haemophili isolates were differentiated based on various observable properties in the growth morphology (e.g. the shape and size of the colony, smooth or rough surface, texture, colony elevation), on a set of biochemical reactions (according to API NH results) and antimicrobial susceptibility results.

Antibiotic sensitivities of *H. parainfluenzae* isolates from patients were determined by the disc diffusion method using Haemophilus Test Medium (HTM, Oxoid, England) according to the Clinical Laboratory Standards Institute (CLSI) recommendation for *Haemophilus* species [28]. Direct colony suspensions standardized to 0.5 McFarland standard (~10^8^ CFU, colony forming units/ml) were prepared using the colonies from an overnight HAEM agar incubation at 35 °C in the atmosphere with about 5 % CO_2_. *H. influenzae* ATCC10211 was used to verify the growth promotion properties of HTM. Different discs with antimicrobial agents (BD BBL, Becton Dickinson and Company, USA), namely ampicillin (10 μg), amoxicillin-clavulanic acid (20/10 μg), ampicillin-sulbactam (10/10 μg), cefazoline (30 μg), cefuroxime (30 μg), cefotaxime (30 μg), ceftazidime (30 μg), imipenem (10 μg), aztreoname (30 μg), azithromycin (15 μg), tetracycline (30 μg), trimethoprim/sulfamethoxazole (1.25/23.75 μg), ciprofloxacin (5 μg) were used. Multidrug resistant haemophili isolates were defined as having resistance to at least three different classes of antimicrobials. Isolates resistant to ampicillin were screened for beta β-lactamase production using *Pen* test (API NH, bioMerieux, France) and the nitrocefin as chromogenic cephalosporin method (Cefinase disks, BD BBL, Becton Dickinson and Company, USA). The test was considered positive if the colour changed from yellow to purple for cephalosporin.

### Statistical analysis

Data processing and analysis were performed using StatSoft, Inc. Statistica 2010 for Windows. Contingency table analysis for comparing proportions was done by Fisher’s exact test. The relative risk (RR) and its 95 % confidence intervals (CI) were calculated. Statistical significance was established at *p* < 0.05.

## Results

In 30/30 (100 %) patients with lung cancer cefuroxime or cefazolin was used during preoperative antimicrobial prophylaxis; in 14/30 (46.7 %) cases it was combined with amikacin. Perioperative prophylaxis alone was applied to 14/30 (46.7 %) patients. Preoperative prophylaxis with postoperative treatment was applied in 16/30 (53.3 %) cases - 15/30 (50 %) patients underwent cefuroxime or cefazolin and 1/30 (3.3 %) patient had beta-lactams with amikacin treatment.

Haemophili were cultured in 21/60 samples taken from the throat and in 0/120 samples taken from the nasal specimens of patients with lung cancer during examinations EI and EII. According to data presented in Table 1, the prevalence of throat colonization by *H. parainfluenzae* in lung cancer patients was higher in EI (19/30, 63.3 %) compared to EII (13/30, 43.3 %). These differences were not statistically significant (*p* = 0.1954). Totally, 20/30 (66.7 %) patients were colonized by *H. parainfluenzae* in both examinations (EI + EII). Besides, *H. influenzae* was isolated from 1/30 (3.3 %) patient (only in EI).

**Table 1:** Frequency of the throat colonization by *Haemophilus parainfluenzae* in healthy people and in patients with lung cancer before (Examination I, EI) and after (Examination II, EII) perioperative prophylaxis without and with postoperative treatment

Group of patients	No. (%) of people			
	Uncolonized by haemophili	Colonized by *Haemophilus parainfluenzae*	RR (95 % CI)	p value
Healthy people (*n* = 65)	16 (24.6)	42 (64.6)	Referent	
Patients with lung cancer (*n* = 30)				
EI	10 (33.3)	19 (63.3)	0.8 (0.4-1.5)	0.6203
EII	17 (56.7)	13 (43.3)	0.5 (0.3-0.8)	0.0106
EI + EII	10 (33.3)	20 (66.7)	0.8 (0.4-1.6)	0.6264

*Abbreviations:* *RR* relative risk

Haemophili were cultured in 49/65 (75.4 %) samples taken from the throat of healthy people. Among 49/65 healthy people colonized by haemophili, 42/65 (64.6 %) cases were colonized by *H. parainfluenzae*, 1/65 (1.5 %) by *H. influenzae* and 6/65 (9.2 %) by other *Haemophilus* spp. Statistically significant differences between the number of healthy people and patients with lung cancer colonized by haemophili were detected in EII (49/65 vs. 13/30, *p* = 0.0048), but not in EI (49/65 vs.20/30, *p* = 0.4591). As was shown in Table 1, statistically significant differences between the number of healthy people and patients with lung cancer colonized by *H. parainfluenzae* were also detected in EII (*p* = 0.0106), but not in EI (*p* = 0.6203).

**Fig. 1:**
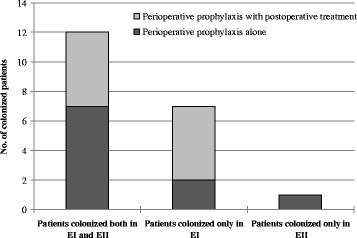
Changes in number of patients with lung cancer colonized by *Haemophilus parainfluenzae* depending on perioperative prophylaxis without or with postoperative treatment on the basis of Examination I (EI) and Examination II (EII) data

Detail analysis of lung cancer patients colonized by *H. parainfluenzae* revealed that 12/30 (40 %) patients were colonized both in EI and EII, 7/30 (23.3 %) only in EI, while 1/30 (3.3 %) patient only in EII (Fig. 1). Among 20 patients colonized totally by *H. parainfluenzae*, in 10/20 (50 %) patients cefazolin or cefuroxime were used only as perioperative prophylaxis, while in 9/20 (45 %) patients cefuroxime and in 1/20 (5 %) patient cefuroxime and amikacin were applied both as preoperative prophylaxis and as postoperative treatment.

Considering the total number of patients with antimicrobial perioperative prophylaxis alone (14/30, 46.7 %) or with antimicrobial perioperative prophylaxis together with postoperative treatment (16/30, 53.3 %) and the number of patients colonized by *H. parainfluenzae* in EI and EII in both groups of patients (Fig. 1), it was shown that perioperative prophylaxis alone did not significantly affected *H. parainfluenzae* prevalence (*p* = 0.7477), while perioperative prophylaxis with postoperative treatment significantly decreased the number of colonized patients (*p* = 0.0626). Moreover, statistically significant differences in *H. parainfluenzae* colonization between healthy people and patients with lung cancer undergoing perioperative prophylaxis with postoperative treatment were also found in EII (*p* = 0.023), but not in patients with perioperative prophylaxis alone (*p* = 0.7609).

According to Table 2, the prevalence of throat colonization by *Candida* spp., mainly *C. albicans*, in lung cancer patients was higher in EII (23/30, 76.7 %) compared to EI (12/30,40 %). *C. albicans* was isolated from 9/30 (30 %) and from 16/30 (53.3 %) patients in EI and EII, respectively. Additionally, 5 of *C. famata* (EI - 1, EII – 4), 4 of *C. glabrata* (EI- 2, EII – 2), and 1 of *C. krusei* (EI- 0, EII – 1) strains were isolated. The differences in the number of patients colonized by *Candida* spp. in EI and in EII were observed (*p* = 0.0082). Additionally, among patients with lung cancer single isolates of Gram-negative rods were cultured (*Escherichia coli*, *Klebsiella pneumoniae*, *Morganella morganii*, *Aeromonas hydrophila, Alcaligenes xylosoxidans*, *Acinetobacter baumannii*, *Kluyvera* spp) mainly in EII.

**Table 2:** Prevalence of throat colonization by *Candida* spp. and other than *Haemophilus* spp.Gram-negative bacteria in patients with lung cancer before (Examination I, EI) and after (Examination II, EII) perioperative prophylaxis without and with postoperative treatment

No. of patient	E I		E II	
	Yeasts	Gram-negative bacteria	Yeasts	Gram-negative bacteria
2	*Candida albicans*	*Klyuvera* spp.	*Candida albicans*	*Klyuvera* spp.
3	*Candida albicans*	-	*Candida albicans*	-
5	-	-	*Candida albicans*	-
6	-	-	*Candida albicans*	-
7	-	-	*Candida famata*	-
8	*-*	*Aeromonas hydrophila*	*Candida albicans*	-
9	-	-	*Candida albicans*	-
11	-	-	*Candida albicans*	*Morganella morganii*
12	*Candida albicans*	-	*Candida albicans*	-
13	-	-	*Candida krusei*	*Alcaligenes xylosoxidans*
14	*-*	-	*Candida famata*	-
15	*Candida albicans*	-	*Candida albicans*	-
16	*Candida albicans*	-	*Candida albicans*	-
17	*Candida glabrata*	-	*Candida glabrata*	*Klebsiella pneumoniae Escherichia coli*
20	*-*	*Escherichia coli*	*-*	*-*
21	*Candida albicans*	-	*Candida albicans* ^a^	-
22	*Candida albicans*	-	*Candida albicans*	-
23	*Candida albicans*	-	*Candida albicans*	*Acinetobacter baumanii*
24	*Candida albicans*	-	*Candida albicans*	-
25		-	*Candida albicans*	-
26	*Candida glabrata Candida famata*	-	*Candida glabrata Candida famata*	-
27	*-*	-	*-*	*Klebsiella pneumoniae*
29	*-*	-	*Candida albicans*	-
30	*-*	-	*Candida famata*	-

^a^Two phenotypically different strains of *Candida albicans* were selected

On the basis of growth morphology, biochemical properties and profile of antimicrobial resistance differences among the number of phenotypically various *H. parainfluenzae* isolates selected from one patient before and after surgery were determined. Totally, 58 *H. parainfluenzae* isolates were cultured from 20 patients with lung cancer – 32/58 (55.2 %) from 19/20 (90 %) patients before (EI) and 26/58 (44.8 %) isolates from 13/20 (65 %) patients after (EII) surgery. Among all selected isolates, 23/58 (39.7 %) were resistant to different antimicrobials – 13/58 (22.4 %) in EI, and 10/58 (17.3 %) in EII (9 isolates in patients without and 1 isolate in a patient with prolonged treatment with antimicrobials). Totally, 13/58 (22.4 %) isolates of *H. parainfluenzae* were resistant to beta-lactams. In the group of patients with preoperative prophylaxis alone, 6/58 (10.3 %) *H. parainfluenzae* isolates both in EI and EII were resistant mainly to beta-lactams (Table 3). Two phenotypically different isolates of *H. parainfluenzae* with increased resistance to antimicrobials were selected in EII only from one patient with cefuroxime as prophylaxis. Among 11/58 (19 %) ampicillin-resistant isolates only three were ampicillin-resistant beta-lactamase positive. Among them 2 isolates were selected both in EI and EII from one patient and therefore it was assumed to be the same strain. Besides, 9 other beta-lactamase negative isolates were resistant to ampicillin and beta-lactams co-administered with a beta-lactamases inhibitor - clavulanic acid or sulbactam. Multidrug resistance (MDR) was detected in 1 isolate, which was resistant to beta-lactams (AmAmcAtm), macrolides (Azm) and trimethoprim/sulphametoxazole (Sxt).

**Table 3:** The effect of antimicrobial preoperative prophylaxis with or without postoperative treatment on the resistance of *Haemophilus parainfluenzae* isolates selected from patients with lung cancer in Examination I (EI) and in Examination II (EII)

Colonized patients			*Haemophilus parainfluenzae* isolates (*n* = 58)			
Number of patients	Prophylaxis	Treatment	Examination	No. (%) of isolates		Profile of resistance (No. of isolates)
				Sensitive	Resistant	
10	Yes	No	EI (*n* = 18)	9 (15.5)	9 (15.5)	Am_Pen+_ (1)
						Te (1)
						Sxt (1)
						SxtTe (1)
						CazCtxCz (2)
						AmAmcSam (1)
						AmAmcCazAtmTe (1)
						AmAmcSamCtxCzAtm (1)
			EII (*n* = 16)	7 (12.1)	9 (15.5)	Am_Pen+_ (1)
						Cz (1)
						Te (1)
						Sxt (1)
						SxtTe (1)
						AmAmcSam (1)
						AmAmcSamCtxCzAtm (1)
						Am_Pen+_SamCazCzCxmCtx (1)
						AmAmcSamCazCtxCzAtmSxt (1)
10	Yes	Yes	EI (*n* = 14)	10 (17.2)	4 (6.9)	Cz (1)
						Sxt (1)
						AmAmcAtmAzmSxt (1)
						AmSamCazCzIpm (1)
			EII (*n* = 10)	9 (15.5)	1 (1.7)	Sxt (1)

*Abbreviations:* *Am* ampicillin; *AmC* amoksicillin/clavulanic acid; *An* amikacin; *Atm* aztreonam; *Azm* azithromycin; *Caz* ceftazidime; *Ctx* cefotaxime; *Cxm* cefuroxime; *Cz* cefazoline; *Ipm* imipenem; *Sxt* trimethoprim/sulfametoksazol; *Te* tetracycline; *Pen +* penicyllinase-positive

## Discussion

It is known that in most cases very strong cancer treatments used today may often affect the health condition and change the defense mechanisms, microbiota conditions and the immune system [29–31]. This treatment, either alone or in combination, usually kills cancer cells and also damages the immune system cells and it can increase the risk of infections or pathogens and opportunistic microbials colonization.

According to our results, the prevalence of haemophili, especially *H. parainfluenzae*, in the airways of investigated patients was relatively stable after a short-term perioperative prophylaxis. This may suggest that perioperative prophylaxis used does not significantly interfere with the microbiota. Conversely, perioperative prophylaxis and postoperative treatment with antibiotics may contribute to both qualitative and quantitative changes within the microbiota represented by *H. parainfluenzae* in our studies. However, it is difficult to define clearly the factors influencing elimination of these bacteria from the airways of patients with lung cancer. In our opinion, the disturbed microbiota composition promotes the mucosa colonization both by the fungi and other bacteria. This was demonstrated mainly in the case of *Candida* spp., but also in Gram-negative bacteria belonging to different species. It could also be the reason for the occurrence of ampicillin-resistant and other beta-lactams resistant isolates or multidrug resistant bacteria during our studies. According to literature, changes in airways colonization in patients with lung cancer after antimicrobial treatment were observed both in nasopharynx samples by Gram-negative rods [32] and in throat samples by *Candida* strains [33]. This may be a precursor to bacterial or fungal respiratory tract infections, which are favoured by, for example, lung cancer and treatment or surgical procedures. Drakulovic et al [34] showed that hospital microflora may colonize patients in the first week of hospitalization and this colonization may increase the severity of the underlying disease and the process of healing after surgery.

Up to 80 % of healthy individuals may carry strains belonging to the genus *Haemophilus* creating nonpathogenic microbiota in the upper respiratory tract [16]. *H. parainfluenzae* is a typical commensal of the indigenous microbiota with unclear pathogenicity in contrast to the accepted pathogenicity of *H. influenzae* [11, 35–37]. These species occasionally, especially when the host‘s immune system is suppressed, may be a cause of localized or even systemic infections [16, 35–38]. Hofstra and co-workers [39] observed the increase of *H. parainfluenzae* in all volunteers during experimental human *Rhinovirus* acute infection. This increase was significant (*p* = 0.0098) but reversible and returned to the baseline level after the infection was cleared. This fact may indicate an important role of these bacteria in the proper functioning of the non-specific immune defence of macroorganisms against colonization by pathogens.

One of undesirable side effects of antibacterial treatment is the deficiency in the nonspecific immune system and colonization of e.g. the mucous membranes with some pathogenic or opportunistic microorganisms [11, 29, 40–43]. However, bacteriological diagnosis depends on the identification of species or their characteristics because the state of microbiota condition is still rare in many laboratories due to the nutritional requirements of these bacteria, the cost and special skills involved.

The effect of both perioperative prophylaxis and a prolonged use of antibiotics on microflora depends on many factors, related both to the type of antimicrobials and to the properties of the microorganisms [27, 40, 44–46]. Beta-lactams, especially cephalosporins, are appropriate first line antimicrobials for most surgical procedures [27, 45]. Perioperative prophylaxis or postsurgical treatment can reduce or even eliminate transient or resident flora and it may be very important as prevention against endogenous infections [25, 41, 42, 47]. It has to be underlined though that they do not protect against pathogens’ colonization. Additionally, in some people this may lead to increased susceptibility to pathogen’s colonization or to opportunistic infections.

This is also compatible with the findings of other authors who noted that disruption of normal microflora may predispose people to infection [8, 26]. Kager et al. [45] investigated microflora in faecal samples of patients who underwent colorectal surgery and received antimicrobial prophylaxis and a prolonged tinidazole administration period, enterococci and streptococci decreased and the number of anaerobic bacteria increased. The authors noted the occurrence of postoperative infections with *E. coli* etiology.

Beta-lactams are the most widely used antibiotics, and beta-lactamases are a greatest source of resistance to them [48]. The phenotypically expressed resistance to beta-lactam antibiotics in haemophili is dependent mainly on the level of production of beta-lactamases and the presence of penicillin-binding protein (PBP) with lowered affinity for these antibiotics as a target site [49–51]. Beta-lactamases were detected in *H. parainfluenzae* isolates from both healthy people and patients with respiratory tract infections. Uraz et al. [52] showed the presence of about 57 % beta-lactamase positive species among the throat cultures of children with upper respiratory tract infections. In literature there is rare information about beta-lactamases and of altered penicillin-binding proteins in *H. parainfluenzae* [51, 53]. According to Gromkova et al. [54, 55], DNA transformation probably plays a major role in the spread of drug resistance in *H. parainfluenzae.* It seems that especially efficient in transformation were the cells classified as biotypes II and I, which are the prevalent biotypes in the world with an ability to develop competence. During natural transformation the transfer of genes occurs via free DNA (from dead or lysed cells) from the surrounding medium by competent bacterial cells [56, 57]. The capacity of absorption of the extracellular DNA by transformation may explain the acquisition of resistance or resistance gene exchange with other bacteria.

## Conclusions

Our results for the first time confirm that antimicrobials prophylaxis together with prolonged postoperative treatment with antimicrobials during short-term hospitalization may disturb the airways microbiota using *H. parainfluenzae* as a marker in lung cancer patients who underwent thoracic surgery. In contrast, neither the disease itself nor short-term hospitalization with perioperative prophylaxis alone significantly affected *H. parainfluenzae* prevalence. In our opinion, haemophili are common and representative bacteria within the upper respiratory tract, and they are a very simple marker of microbiota condition with regard to their role as a protective factor for the host organism. Additionally, a study of the respiratory microbiota composition and its disturbance may unfold new insights and approaches to the pathogenesis of lung diseases.

### Ethics approval

The Ethics Committee of the Medical University of Lublin approved study protocol (KE-0254/75/2011).

### Consent statement

All participants, including the healthy volunteers, gave a conscious oral consent to take part in the studies.

### Availability of data and materials

Data presented in this study are complete. No supplementary files are attached.

## Abbreviations

EI: Examination I
EII: Examination II
HAEM: *Haemophilus* chocolate agar
HTM: *Haemophilus* test medium
TSA: Tripticasein Soy Lab-Agar
